# Clinical Phenotypes With Prognostic Implications in Pulmonary Embolism Patients With Syncope

**DOI:** 10.3389/fcvm.2022.836850

**Published:** 2022-02-15

**Authors:** Shuai Zhang, Xiaomao Xu, Yingqun Ji, Yuanhua Yang, Qun Yi, Hong Chen, Xiaoyun Hu, Zhihong Liu, Yimin Mao, Jie Zhang, Juhong Shi, Jieping Lei, Dingyi Wang, Zhu Zhang, Sinan Wu, Qian Gao, Xincao Tao, Wanmu Xie, Jun Wan, Yunxia Zhang, Meng Zhang, Xiang Shao, Zhonghe Zhang, Baomin Fang, Peiran Yang, Zhenguo Zhai, Chen Wang, the China pUlmonary Thromboembolism REgistry Study (CURES) Investigators

**Affiliations:** ^1^Department of Pulmonary and Critical Care Medicine, Center of Respiratory Medicine, China-Japan Friendship Hospital, Beijing, China; ^2^National Center for Respiratory Medicine, Beijing, China; ^3^Institute of Respiratory Medicine, Chinese Academy of Medical Sciences, Beijing, China; ^4^National Clinical Research Center for Respiratory Disease, Beijing, China; ^5^Department of Pulmonary and Critical Care Medicine, Beijing Hospital, Beijing, China; ^6^Department of Pulmonary and Critical Care Medicine, The First Affiliated Hospital of Dalian Medical University, Dalian, China; ^7^Department of Pulmonary and Critical Care Medicine, Beijing Chao-Yang Hospital, Capital Medical University, Beijing, China; ^8^Department of Pulmonary and Critical Care Medicine, West China Hospital, West China School of Medicine, Sichuan University, Chengdu, China; ^9^Department of Pulmonary and Critical Care Medicine, The First Affiliated Hospital of Chongqing Medical University, Chongqing, China; ^10^Department of Pulmonary and Critical Care Medicine, First Hospital of Shanxi Medical University, Taiyuan, China; ^11^National Center for Cardiovascular Diseases, Fuwai Hospital, Chinese Academy of Medical Science, Beijing, China; ^12^Department of Pulmonary and Critical Care Medicine, The First Affiliated Hospital of Henan University of Science and Technology, Luoyang, China; ^13^Department of Pulmonary and Critical Care Medicine, The Second Hospital of Jilin University, Changchun, China; ^14^Department of Pulmonary and Critical Care Medicine, Peking Union Medical College Hospital, Beijing, China; ^15^Data and Project Management Unit, Institute of Clinical Medical Sciences, China-Japan Friendship Hospital, Beijing, China; ^16^Department of Pulmonary and Critical Care Medicine, Beijing Anzhen Hospital, Capital Medical University, Beijing, China; ^17^Department of Traditional Chinese Medicine for Pulmonary Diseases, Center of Respiratory Medicine, China-Japan Friendship Hospital, Beijing, China; ^18^Department of Pathophysiology, Peking Union Medical College, Beijing, China; ^19^Peking Union Medical College, Chinese Academy of Medical Sciences, Beijing, China; ^20^Department of Respiratory Medicine, Capital Medical University, Beijing, China

**Keywords:** pulmonary embolism, syncope, mortality, phenotype, cluster analysis

## Abstract

**Objectives:**

There are conflicting data concerning the prognostic significance of syncope in acute pulmonary embolism (PE). This study aimed to investigate the impact of syncope on clinical outcomes of acute PE, and determine the clinical phenotypes of PE patients with syncope and their correlation with prognosis.

**Methods:**

In the ongoing, national, multicenter, registry study, the China pUlmonary thromboembolism REgistry Study (CURES) enrolling consecutive patients with acute PE, patients with and without syncope were investigated. Principal component analysis (PCA) was performed using nine variables relevant to syncope and PE, including age, sex, body mass index, history of cardiovascular disease, recent surgery or trauma, malignancy, pulse, systolic blood pressure, and respiratory rate. Patient classification was performed using cluster analysis based on the PCA-transformed data. The clinical presentation, disease severity and outcomes were compared among the phenotypes.

**Results:**

In 7,438 patients with acute PE, 777 (10.4%) had syncope, with younger age, more females and higher body mass index. Patients with syncope had higher frequency of precordial pain, palpitation, and elevated cardiac biomarkers, as well as higher D-Dimer level. In the syncope group, more patients had right ventricular/left ventricular ratio > 0.9 in ultrasonic cardiogram and these patients had higher estimated pulmonary arterial systolic pressure compared with patients without syncope. As the initial antithrombotic treatment, more patients with syncope received systemic thrombolysis. Despite a higher prevalence of hemodynamic instability (OR 7.626, 95% CI 2.960–19.644, *P* < 0.001), syncope did not increase in-hospital death. Principal component analysis revealed that four independent components accounted for 60.3% of variance. PE patients with syncope were classified into four phenotypes, in which patients with high pulse and respiratory rate had markedly higher all-cause mortality during admission.

**Conclusion:**

Syncope was associated with hemodynamic instability and more application of thrombolysis, without increasing in-hospital deaths. Different clinical phenotypes existed in PE patients with syncope, which might be caused by various mechanisms and thus correlated with clinical outcomes.

## Introduction

High morbidity and poor clinical outcomes associated with pulmonary embolism (PE) require accurate and rapid risk assessment for patients. So far, risk stratification is mainly based on the presence of hemodynamic instability, right ventricular dysfunction (RVD) and myocardial injury (1). Clinical presentation of PE varies widely from hemodynamic instability to clinically silent disease, incidentally discovered on computed tomography or found on autopsy of patients with unexpected sudden death (2, 3). Typical symptoms of acute PE had been reported to be associated with adverse outcome (4, 5). Syncope presents as one of the initial symptoms in 9–35% of acute PE cases (6–11). Conversely, the incidence of objectively confirmed acute PE was 1.4–17.3% in the patients hospitalized for a first episode of syncope (12–15).

There are conflicting data concerning the prognostic significance of syncope in patients with acute PE. Several studies suggested that syncope is associated with higher mortality (11, 16). In the ICOPER registry, the 3-month mortality of patients with syncope was 26.8%, significantly higher than the overall mortality of 17% (17). Syncope has been used in combination with cardiac biomarkers and tachycardia to develop a model for advanced risk stratification in PE (18, 19). However, many studies did not reproduce the association between syncope and higher mortality (6, 7, 9, 10, 20, 21). Syncope may occur in the presence or absence of hemodynamic instability, the mechanism of which is not clear. Different clinical phenotypes may exist in PE patients with syncope, with heterogeneous pathogenesis. Identification of the relatively higher-risk group among PE patients with syncope could improve the risk stratification and prognosis of these patients.

As an ongoing, national, multicenter, registry study, the China pUlmonary thromboembolism REgistry Study (CURES) enrolls consecutive patients diagnosed with acute symptomatic PE. We analyzed data in the CURES registry to explore the impact of syncope on the characteristics, therapeutic strategy and outcomes in patients with confirmed acute PE. Clinical phenotypes of PE patients with syncope were determined and their impact on clinical outcomes were further identified.

## Materials and Methods

### Patients Enrollment

The CURES registry is an ongoing national, multicenter, observational, prospective registry study, involving 100 medical centers across China. Consecutive patients greater than or equal to 18 years and diagnosed with acute symptomatic PE have been enrolled since 2009. In the patients with suspected PE, computed tomographic pulmonary angiography, ventilation-perfusion lung scintigraphy, magnetic resonance pulmonary angiography or pulmonary angiography were used to confirm the diagnosis. Patients were managed according to the clinical practice of each participating hospital center.

The registry complies with the Declaration of Helsinki and was approved by ethics committees in participating centers and hospital-based institutional review boards. The CURES registry is registered on ClinicalTrials.gov (NCT02943343). All patients provided written informed consent for their participation in the registry, in accordance with the requirements of the ethics committee in each hospital.

### Data Collection

Data were recorded in a standardized case report form based on the original medical records at each participating center. Patients enrolled in CURES had data collected that included demographic information, comorbidities, risk factors for PE, symptoms and signs, physical and laboratory examinations, in addition to results of image testing, therapeutic management and clinical outcomes both in hospital and during the follow-up. Data quality was regularly monitored and documented electronically to identify inconsistencies or errors, which were resolved by the local coordinators at each participating center.

### Variable Definition and Clinical Endpoint

Patients were allocated into two groups based on the presence of syncope, defined as a sudden transient loss of consciousness that has a rapid onset, short duration and spontaneous resolution (22). The primary endpoint was all-cause death and fatal PE during admission. For deaths confirmed by autopsy or those following a clinically severe PE, in the absence of any alternative diagnosis, the investigators were instructed to judge if the death was due to fatal PE. Major bleeding was defined as previously reported (23). Death, cause of death and major bleeding events were adjudicated by the registry coordinators.

Hemodynamic instability, also defined as high-risk, was defined according to the European Society of Cardiology Guidelines (1). Simplified pulmonary embolism severity index (sPESI) was calculated (24) for patients with hemodynamically stable PE. Cardiac biomarkers used in the risk assessment of PE include cardiac troponin I, cardiac troponin T, brain natriuretic peptide (BNP), and N terminal-pro BNP (NT-proBNP).

### Statistical Analyses

Qualitative data were reported as *n* (%). Quantitative data were reported as mean (standard deviation, SD) or median (interquartile range, IQR). Independent *T*-tests and one-way ANOVA were used to compare mean of normally distributed data, while nonparametric tests were used to compare non-normally distributed or discrete data. The χ^2^ test was used to compare categorical data. A logistic regression model was used to identify the risk factors for syncope. Any variable achieving a *P*-value < 0.1 on univariate analysis was included in a multivariate logistic regression analysis. Odds ratio (OR) and the corresponding 95% confidence interval (CI) were reported. Nine variables were selected for their relevance to syncope and PE: age, sex, body mass index (BMI), history of cardiovascular disease (CVD), recent surgery or trauma, malignancy, pulse, systolic blood pressure (SBP), and respiratory rate (RR). On account of redundancy, principal component analysis (PCA) was performed on these variables to reduce interaction between variables. Then, cluster analysis was performed based on the main principal components to identify phenotype clusters of syncope in PE patients. The clinical characteristics and outcomes were compared among these phenotype clusters. A *P*-value of <0.05 was considered to be statistically significant. All the analyses were performed using the IBM SPSS software (Version 26.0).

## Results

**Figure 1:**
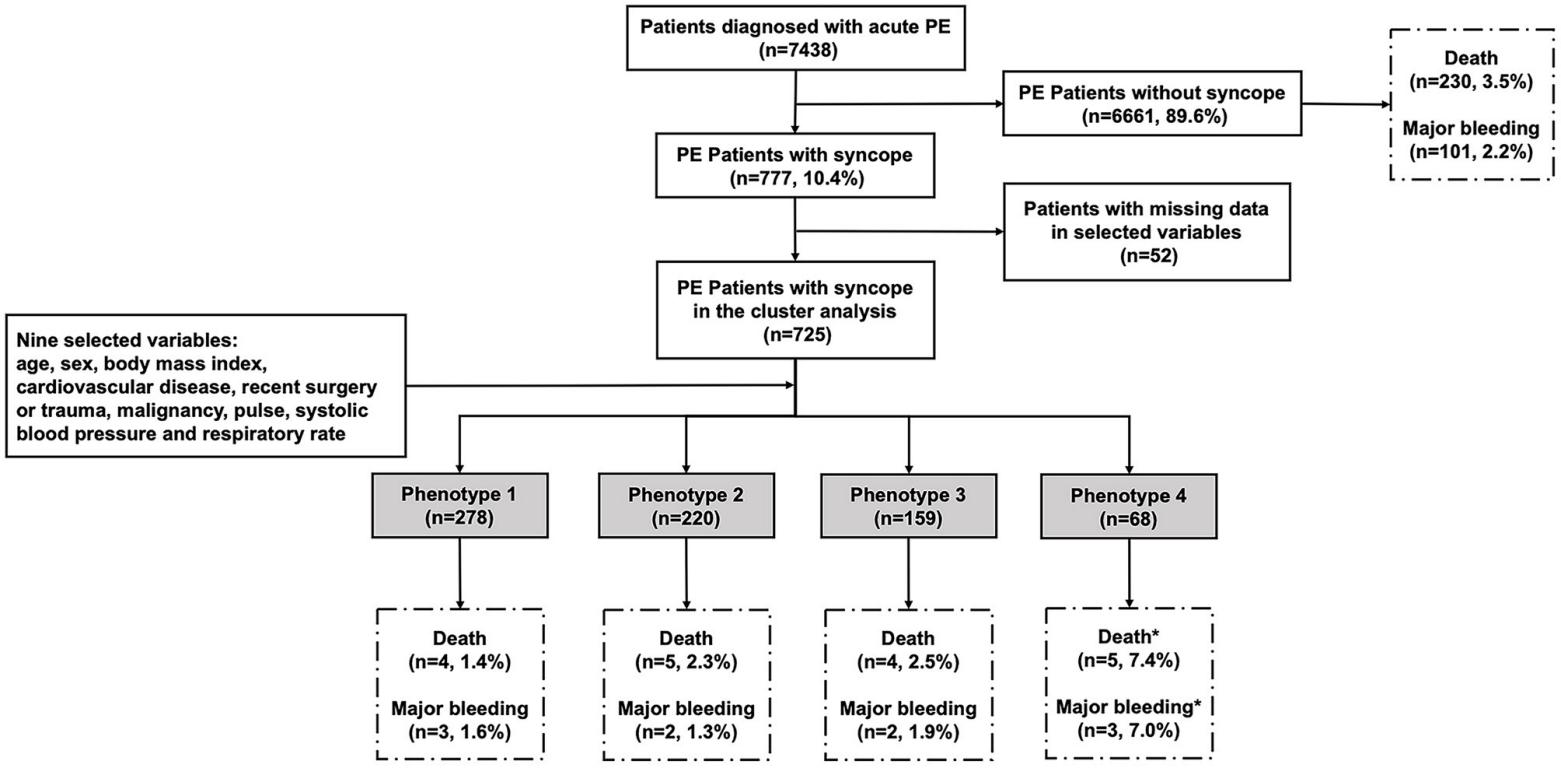
The flowchart of the study. We enrolled 7,438 patients confirmed with acute PE, in which 777 patients had syncope as one of initial symptoms. Nine variables were selected for their relevance to syncope and PE: age, sex, body mass index, cardiovascular disease, recent surgery or trauma, malignancy, pulse, systolic blood pressure, and respiratory rate. Complete data for the nine variables were available for 725 subjects with syncope. In the system cluster analysis, they were classified into four phenotypes, with different outcomes during admission. ^*^The difference is statistically significant compared with other phenotypes.

Between January 2009 and December 2015, a total of 7,438 consecutive adult patients with acute PE were included in the CURES registry. Of these patients 777 (10.4%) had syncope. A flowchart describing the main methodology and results of this study is shown in Figure 1.

### Demographic Characteristics and Comorbidities

Of all the patients, 3,939 (53.0%) are males, while there were fewer males in patients with syncope (43.2 vs. 54.1%, *P* < 0.001). The mean age of all patients was 61.3 ± 15.1 years and patients with syncope were younger than those without (60.1 ± 14.9 years vs. 61.4 ± 15.1 years, *P* = 0.023). Patients with syncope had higher BMI (24.4 ± 3.5 kg/m^2^ vs. 24.0 ± 3.6 kg/m^2^, *P* = 0.012) (Table 1).

**Table 1:** Demographic characteristics, comorbid diseases, and risk factors of patients with acute PE.

**Characteristics**	**Patients with syncope**	**Patients without syncope**	***P-*value**
	**(*n* = 777, 10.4%)**	**(*n* = 6,661, 89.6%)**	
**Demographic characteristics**			
Age, year, mean ± SD	60.1 ± 14.9	61.4 ± 15.1	0.023^*^
Male, *n* (%)	336 (43.2)	3,603 (54.1)	<0.001^*^
BMI, kg/m^2^, mean ± SD	24.4 ± 3.5	24.0 ± 3.6	0.012^*^
**Comorbid diseases**, ***n*** **(%)**			
Cardiovascular diseases			
Hypertension	296 (38.1)	2,374 (35.7)	0.180
Coronary heart disease	96 (12.4)	908 (13.6)	0.322
Rheumatic heart disease	4 (0.4)	44 (0.7)	0.807
Cardiomyopathy	3 (0.4)	42 (0.6)	0.557
Heart failure	18 (2.3)	344 (5.2)	<0.001^*^
Respiratory diseases			
Chronic obstructive pulmonary disease	38 (4.9)	560 (8.4)	0.001^*^
Pulmonary infection	47 (6.0)	813 (12.2)	<0.001^*^
Tuberculosis	20 (2.6)	206 (3.1)	0.424
Asthma	6 (0.8)	90 (1.4)	0.176
Interstitial lung disease	9 (1.2)	121 (1.8)	0.185
Bronchiectasis	4 (0.5)	87 (1.3)	0.084
Cor pulmonale	21 (2.7)	168 (2.5)	0.764
Diabetes mellitus	76 (9.8)	719 (10.8)	0.400
Neurological diseases			
Ischemic stroke	66 (8.5)	578 (8.7)	0.856
Hemorrhagic stroke	10 (1.3)	129 (1.9)	0.204
Liver and kidney diseases			
Chronic hepatitis	10 (1.3)	117 (1.8)	0.340
Cirrhosis	5 (0.6)	23 (0.3)	0.199
Chronic nephritis	5 (0.6)	65 (1.0)	0.365
Nephrotic syndrome	9 (1.2)	76 (1.1)	0.964
Varicose veins	75 (9.7)	466 (7.0)	0.007^*^
**Risk factors for PE**, ***n*** **(%)**			
Malignancy	69 (8.9)	830 (12.5)	0.004^*^
Surgery in recent 3 months	113 (14.6)	895 (13.5)	0.398
Trauma in recent 3 months	61 (7.9)	541 (8.2)	0.769
Central venous catheterization	5 (0.7)	37 (0.6)	0.770
Oral contraceptives	7 (0.9)	19 (0.3)	0.006^*^
Pregnancy	3 (0.4)	80 (1.3)	0.060
Postpartum	73 (9.8)	523 (8.2)	0.152
**Smoking**, ***n*** **(%)**			
Ever or current smokers	192 (30.8)	2067 (37.5)	0.001^*^

*PE, pulmonary embolism; SD, standard deviation; BMI, body mass index*.

* *The difference is statistically significant*.

There were significantly fewer patients with heart failure, chronic obstructive pulmonary disease and pulmonary infection in the syncope group. Compared with patients without syncope, more patients with syncope had varicose veins (9.7 vs. 7.0%, *P* = 0.007), while fewer patients had malignancy (8.9 vs. 12.5%, *P* = 0.004).

### Clinical Presentation and Risk Stratification

Patients with syncope had higher frequency of precordial pain and palpitation, whereas cough, sputum, fever, pleurisy pain and hemoptysis were more common in patients without syncope. Compared with the non-syncope group, there were more patients with pulse ≥ 110 beats/min, RR > 20 breath/min and SBP < 100 mmHg in the syncope group (*P* < 0.001, *P* = 0.001, *P* < 0.001) (Table 2).

**Table 2:** Clinical presentation, initial treatment, and clinical outcomes of patients with acute PE.

**Characteristics**	**Patients with syncope**	**Patients without syncope**	***P-*value**
	**(*n* = 777)**	**(*n* = 6,661)**	
**Symptoms**, ***n*** **(%)**			
Cough	220 (28.3)	2,888 (43.5)	<0.001^*^
Sputum	171 (22.0)	2,222 (33.4)	<0.001^*^
Fever	60 (7.7)	1,065 (16.0)	<0.001^*^
Dyspnea	541 (69.6)	4,452 (67.0)	0.139
Precordial pain	243 (31.3)	1,594 (24.0)	<0.001^*^
Pleurisy pain	93 (12.0)	1,193 (18.0)	<0.001^*^
Hemoptysis	62 (8.0)	943 (14.2)	<0.001^*^
Palpitation	193 (24.8)	807 (12.1)	<0.001^*^
**Signs**			
Temperature, °C, median (IQR)	36.5 (36.2, 36.8)	36.5 (36.3, 36.9)	<0.001^*^
Pulse ≥ 110 beats/min, *n* (%)	104 (13.6)	634 (9.6)	<0.001^*^
RR > 20 breath/min, *n* (%)	323 (41.8)	2,357 (35.6)	0.001^*^
SBP < 100 mmHg, *n* (%)	77 (10.0)	291 (4.4)	<0.001^*^
Shock index>1, *n* (%)	77 (10.1)	337 (5.1)	<0.001^*^
**Laboratory findings**			
WBC > 10 ×10^9^/L, *n* (%)	240 (31.4)	1,697 (25.9)	0.001^*^
Anemia, *n* (%)	150 (19.7)	1,459 (22.5)	0.081
Platelet < 100 ×10^9^/L, *n* (%)	63 (8.3)	366 (5.6)	0.003^*^
PaO_2_ < 60 mmHg, *n* (%)	157 (22.1)	1,160 (20.4)	0.301
eGFR < 60 mL/min/1.73 m^2^, *n* (%)	122 (16.4)	831 (13.1)	0.015^*^
Elevated cardiac biomarkers, *n* (%)	410 (52.8)	2,328 (34.9)	<0.001^*^
D-Dimer, μg/L, median (IQR)	1,391.4 (474.0, 4,050.8)	1,029.0 (362.0, 3,160.0)	<0.001^*^
**Risk stratification**, ***n*** **(%)**			
Hemodynamically unstable	116 (14.9)	194 (2.9)	<0.001^*^
Hemodynamically stable	661 (85.1)	6,467 (97.1)	<0.001^*^
sPESI ≥ 1	463 (70.0)	4,528 (70.0)	0.998
sPESI = 0	198 (30.0)	1,939 (30.0)	0.998
**Initial treatment**, ***n*** **(%)**			
Anticoagulation	563 (72.5)	5,664 (85.0)	<0.001^*^
Systemic thrombolysis	166 (21.4)	529 (7.9)	<0.001^*^
IVC filter transplantation	33 (4.4)	353 (5.5)	0.204
Interventional thrombectomy	4 (0.5)	22 (0.3)	0.619
Surgical embolectomy	5 (0.7)	46 (0.7)	0.881
**In-hospital Outcomes**, ***n*** **(%)**			
Death	24 (3.1)	230 (3.5)	0.597
Fatal PE	16 (2.1)	102 (1.5)	0.265
Major bleeding	12 (2.3)	101 (2.2)	0.879
**Length of stay, days, median (IQR)**	13 (9, 19)	14 (9, 19)	0.733

*PE, pulmonary embolism; RR, respiratory rate; SBP, systolic blood pressure; WBC, white blood cell; eGFR, estimated glomerular filtration rate, assessed by CKD-EPI formula; sPESI, simplified pulmonary embolism severity index; IVC, inferior vena cava; IQR, interquartile range*.

* *The difference is statistically significant*.

Patients with syncope were more likely to have white blood cell > 10×10^9^/L (31.4 vs. 25.9%, *P* = 0.001) and platelet < 100×10^9^/L (8.3 vs. 5.6%, *P* = 0.003). In the syncope group, more patients had elevated cardiac biomarkers, including cardiac troponin, BNP and NT-proBNP (52.8 vs. 34.9%, *P* < 0.001). There were also more patients with estimated glomerular filtration rate (eGFR) < 60 mL/min/1.73 m^2^ in the syncope group (16.4 vs. 13.1%, *P* = 0.015). The D-Dimer level of patients with syncope was significantly higher compared with that of patients without syncope (1,391.4 vs. 1,029.0 μg/L, *P* < 0.001) (Table 2).

The electrocardiogram (ECG) and cardiac ultrasonography (UCG) findings were also presented in Supplementary Table 1, as well as thrombus location in CTPA. In the syncope group, there were more patients with S_I_Q_III_T_III_ in ECG, more patients with RV/LV ratio > 0.9 and RV free wall mobility ≤ 5 mm in UCG. These patients also had higher level of estimated pulmonary arterial systolic pressure compared with the non-syncope group. More patients had thrombus in central pulmonary arteries (including pulmonary trunk, right and left pulmonary artery) in the syncope group.

Of the 7,438 patients enrolled, 310 (4.2%) patients had hemodynamically unstable PE. The proportion of patients with hemodynamically unstable PE were markedly higher in the syncope group than that in the non-syncope group (14.9 vs. 2.9%, *P* < 0.001). In patients with hemodynamically stable PE, there was no significant difference in the sPESI classification between the two groups.

Further comparison of the demographic characteristics, comorbidities and clinical presentation was performed between patients with and without syncope in hemodynamic stable and unstable groups, and the results were listed in Supplementary Table 2.

### Initial Anti-thrombotic Therapy

Regarding initial treatment, 6,227 (83.7%) patients received anticoagulation, while 695 (9.3%) patients received systemic thrombolysis. Compared to patients without syncope, more patients received thrombolysis (21.4 vs. 7.9%, *P* < 0.001) in the syncope group (Table 2).

In the further analysis of the hemodynamically unstable PE patients, 51.7% of syncope patients and 35.1% of non-syncope patients received systemic thrombolysis (*P* = 0.004). There were also more patients with syncope who received thrombolysis among the hemodynamically stable PE patients (16.0 vs. 7.1%, *P* < 0.001) (Supplementary Table 3).

### In-hospital Outcomes

Of all the patients, 254 (3.4%) patients died and 118 (1.6%) patients had fatal PE, while 113 patients (2.2%) had major bleeding. Clinical outcomes in PE patients with and without syncope was shown in Table 2. There was no significant difference between patients with and without syncope with regard to the incidence of all-cause mortality, fatal PE, major bleeding, or length of stay.

### Clinical Factors Related With Syncope

**Figure 2:**
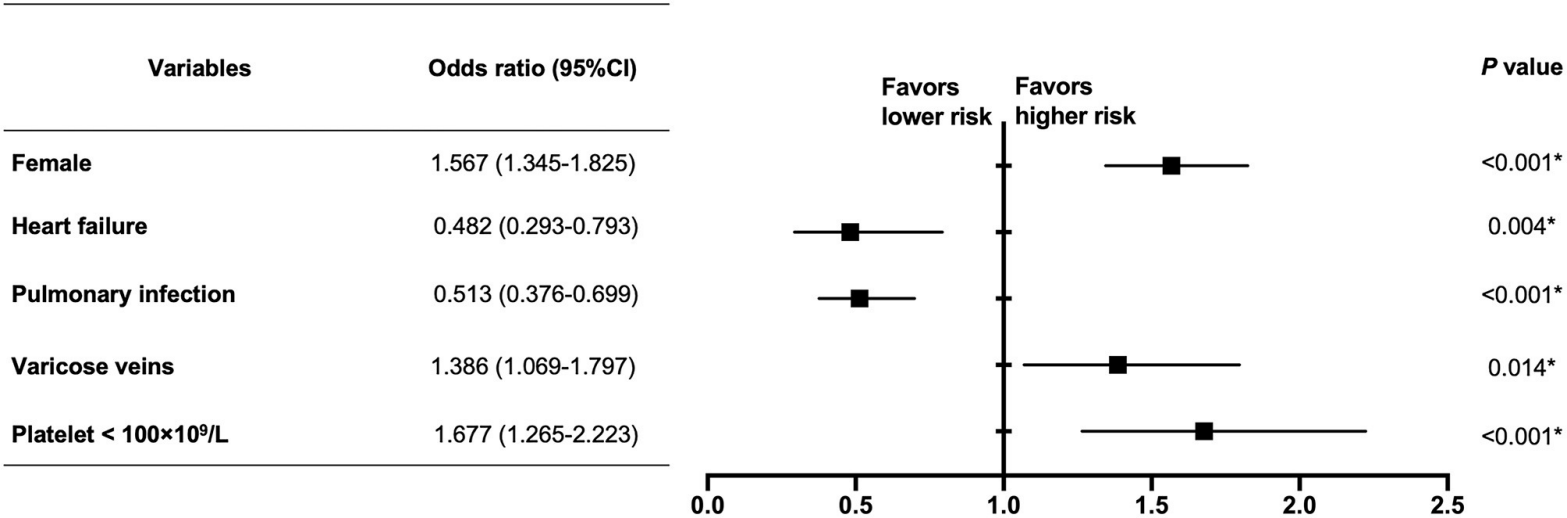
Multivariate analysis of clinical factors related with syncope in PE patients. In the multivariate analysis, female (OR 1.567, 95% CI 1.345–1.825, *P* < 0.001), varicose veins (OR 1.386, 95% CI 1.069–1.797, *P* = 0.014), and platelet < 100 × 10^9^/L (OR 1.677, 95% CI 1.265–2.223, *P* < 0.001) were independent risk factors related with syncope in PE, while heart failure (OR 0.482, 95% CI 0.293–0.793, *P* = 0.004) and pulmonary infection (OR 0.513, 95% CI 0.376–0.699, *P* < 0.001) were protective factors. ^*^The difference is statistically significant.

In the multivariate analysis, female (OR 1.567, 95% CI 1.345–1.825, *P* < 0.001), varicose veins (OR 1.386, 95% CI 1.069–1.797, *P* = 0.014) and platelet < 100×10^9^/L (OR 1.677, 95% CI 1.265–2.223, *P* < 0.001) were independent risk factors related with syncope in PE, while history of heart failure (OR 0.482, 95% CI 0.293–0.793, *P* = 0.004) and pulmonary infection (OR 0.513, 95% CI 0.376–0.699, *P* < 0.001) were protective factors (Figure 2).

### PCA of Clinical Variables

Complete data for the nine variables, necessary for principal component and cluster analyses, were available for 725 subjects with syncope. PCA was performed to transform data in the nine selected variables into four independent components, which contributed significantly to explaining the relationships among the selected variables (eigenvalues > 1) accounted for 60.3% of the variance. Correlations of the selected variables with these four independent components are shown in Table 3. Component 1 was correlated with age, CVD and SBP, and was inversely correlated with pulse. Component 2 was correlated with pulse and RR. Component 3 was correlated with recent surgery or trauma and malignancy, and was inversely correlated with sex. Component 4 was correlated with BMI and recent surgery or trauma.

**Table 3:** Correlations of the nine original variables with the four main components derived from the principal component analysis in the 725 PE patients with syncope.

	**Components**			
	**1**	**2**	**3**	**4**
Sex	0.070	0.188	−0.363	0.237
Age	0.645	0.328	0.273	−0.162
Body mass index	−0.088	0.224	−0.320	0.764
Cardiovascular disease	0.599	0.541	−0.017	−0.062
Recent surgery or trauma	−0.250	0.071	0.575	0.505
Malignancy	−0.151	0.158	0.754	0.065
Pulse	−0.595	0.570	−0.072	−0.140
Systolic blood pressure	0.568	0.293	0.007	0.232
Respiratory rate	−0.466	0.673	−0.091	−0.242

*The variance of components 1, 2, 3, and 4 were 19.6, 15.3, 13.6, and 11.8%, respectively*.

### Clusters of Patients With Syncope

In order to classify of PE subjects with syncope, the four principal components identified above were used in a cluster analysis. Pseudo-F and pseudo-t^2^ statistics determined that the data could be optimally grouped into four clusters. Clinical characteristics of the 725 PE patients with syncope according to these four phenotypes clusters were presented in Table 4.

**Table 4:** Characteristics of the 725 PE patients with syncope according to the four phenotypes identified using principal component analysis-based cluster analysis.

	**Cluster 1**	**Cluster 2**	**Cluster 3**	**Cluster 4**	***P*-value**
	**(*****n*** **= 278)**	**(*****n*** **= 220)**	**(*****n*** **= 159)**	**(*****n*** **= 68)**	
**Demographic characteristics**					
Age, years, mean ± SD	54.3 ± 14.6	67.9 ± 10.9	59.4 ± 15.7	60.8 ± 13.1	<0.001^*^
Male, *n* (%)	136 (48.9)	67 (30.5)	80 (50.3)	29 (42.6)	<0.001^*^
BMI, kg/m^2^, mean ± SD	23.6 ± 3.0	25.2 ± 3.8	24.5 ± 3.6	24.9 ± 4.1	<0.001^*^
**Comorbid diseases**, ***n*** **(%)**					
CVD	41 (14.7)	183 (83.2)	53 (33.3)	43 (63.2)	<0.001^*^
Respiratory diseases	42 (15.1)	31 (14.1)	19 (11.9)	11 (16.2)	0.785
Neurological diseases	14 (5.1)	33 (15.0)	12 (7.5)	7 (10.6)	0.002^*^
Diabetes mellitus	17 (6.2)	33 (15.1)	17 (10.7)	7 (10.4)	0.014^*^
Liver and kidney diseases	9 (3.2)	9 (4.1)	4 (2.5)	2 (2.9)	0.858
**Risk factors for PE**, ***n*** **(%)**					
Recent surgery or trauma	0	0	124 (78.0)	21 (30.9)	<0.001^*^
Malignancy	0	0	57 (35.8)	9 (13.2)	<0.001^*^
**Symptoms and signs**, ***n*** **(%)**					
Fever	22 (7.9)	9 (4.1)	18 (11.3)	7 (10.3)	0.055
Cough	73 (26.3)	62 (28.2)	43 (27.0)	31 (45.6)	0.015^*^
Dyspnea	196 (70.5)	140 (63.6)	116 (73.0)	53 (77.9)	0.075
Chest pain	113 (40.6)	84 (38.2)	56 (35.2)	23 (33.8)	0.604
Hemoptysis	34 (12.2)	11 (5.0)	10 (6.3)	4 (5.9)	0.016^*^
Palpitation	68 (24.5)	49 (22.3)	33 (20.8)	27 (39.7)	0.016^*^
Pulse ≥ 110 beats/min	32 (11.5)	8 (3.6)	17 (10.7)	40 (58.8)	<0.001^*^
SBP < 100 mmHg	37 (13.3)	3 (1.4)	21 (13.2)	10 (14.7)	<0.001^*^
RR > 20 breath/min	112 (40.3)	65 (29.5)	52 (32.7)	68 (100.0)	<0.001^*^
**Laboratory findings**					
WBC > 10×10^9^/L, *n* (%)	89 (32.5)	42 (19.7)	51 (32.1)	35 (51.5)	<0.001^*^
Anemia, *n* (%)	53 (19.5)	26 (12.2)	45 (28.3)	13 (19.1)	0.002^*^
Platelet < 100×10^9^/L, *n* (%)	33 (12.1)	9 (4.2)	11 (6.9)	5 (7.4)	0.016^*^
Elevated cardiac biomarkers, *n* (%)	151 (54.3)	112 (50.9)	81 (50.9)	41 (60.3)	0.513
PaO_2_ < 60 mmHg, *n* (%)	53 (20.9)	40 (19.9)	26 (17.9)	26 (40.6)	0.002^*^
eGFR < 60 mL/min/1.73 m^2^, *n* (%)	29 (11.2)	40 (18.8)	23 (14.8)	18 (26.9)	0.008^*^
D-Dimer, μg/L, median (IQR)	1,247.0 (425.8, 3,915.0)	1,201.0 (442.0, 3,502.2)	1,539.0 (758.5, 4,671.0)	2,338.0 (585.0, 6,455.0)	0.033^*^
**Risk stratification**, ***n*** **(%)**					
Hemodynamically unstable	40 (14.4)	19 (8.6)	27 (17.0)	18 (26.5)	0.002^*^
Hemodynamically stable	238 (85.6)	201 (91.4)	132 (83.0)	50 (73.5)	0.002^*^
sPESI ≥ 1	126 (52.9)	173 (86.1)	92 (69.7)	45 (90.0)	<0.001^*^
sPESI = 0	112 (47.1)	28 (13.9)	40 (30.0)	5 (10.0)	<0.001^*^
**Initial treatment**, ***n*** **(%)**					
Anticoagulation	198 (71.2)	175 (79.5)	123 (77.4)	37 (54.4)	<0.001^*^
Systemic thrombolysis	67 (24.1)	37 (16.8)	26 (16.4)	23 (33.8)	0.005^*^
IVC filter implantation	14 (5.2)	5 (2.3)	9 (5.8)	5 (7.6)	0.202
**In-hospital outcomes**, ***n*** **(%)**					
Death	4 (1.4)	5 (2.3)	4 (2.5)	5 (7.4)	0.047^*^
Fatal PE	1 (0.4)	3 (1.4)	3 (1.9)	4 (5.9)	0.010^*^
Major bleeding	3 (1.6)	2 (1.3)	2 (1.9)	3 (7.0)	0.115
**Length of stay, days, median (IQR)**	13 (9, 18)	14 (10, 20)	14 (9, 20)	13 (9, 21)	0.303

*PE, pulmonary embolism; SD, standard deviation; BMI, body mass index; CVD, cardiovascular disease; IQR, interquartile range; SBP, systolic blood pressure; RR, respiratory rate; WBC, white blood cell; eGFR, estimated glomerular filtration rate, assessed by CKD-EPI formula; sPESI, simplified pulmonary embolism severity index; IVC, inferior vena cava*.

* *The difference is statistically significant*.

We found marked differences among these groups. Phenotype B was composed of older subjects (*n* = 220, mean age 67.9 years) and more females (69.5%) with higher BMI and frequent CVD. No patients had recent surgery or trauma, or malignancy as risk factors for PE in this phenotype. Compared with the other three phenotypes, phenotype B was significantly less likely to have SBP < 100 mmHg, while no significant difference was found in the prevalence of low SBP (< 100 mmHg) among phenotypes A, C and D. Phenotype A had relatively young subjects (*n* = 278, mean age 54.3 years), with no patients with recent surgery or trauma, or malignancy, and CVD was infrequent. On the contrary, phenotype C had high prevalence of recent surgery or trauma (78.0%) and malignancy (35.8%). Phenotype D had significantly higher prevalence of pulse ≥ 110 beats/min and RR > 20 breath/min. Furthermore, the proportion of patients with PaO_2_ < 60 mmHg and eGFR < 60 mL/min/1.73 m^2^ as well as the D-Dimer level were markedly higher in phenotype D, which also had more patients with hemodynamically unstable PE and sPESI ≥ 1. These four phenotypes were summarized in Table 5.

**Table 5:** Summary of syncope phenotypes identified using principal component analysis-based cluster analysis.

	**Phenotype A:**	**Phenotype B:**	**Phenotype C:**	**Phenotype D:**
	**Young/unprovoked PE**	**Old/female/CVD/high BMI and SBP**	**Recent surgery or trauma/malignancy**	**High pulse and RR**
Age	Young	Old	-	-
Sex	-	Female	-	-
BMI	Normal	High	Normal	Normal
CVD	Infrequent	Very frequent	Less frequent	Frequent
Recent surgery or trauma	None	None	Very frequent	Frequent
Malignancy	None	None	Frequent	Less frequent
Pulse and RR	Normal	Normal	Normal	High
SBP	Normal	High	Normal	Normal

*PE, pulmonary embolism; BMI, body mass index; CVD, cardiovascular disease; RR, respiratory rate; SBP, systolic blood pressure*.

### Impact of Phenotypes on Outcomes

The comparison of in-hospital outcomes and length of stay among these four phenotypes were presented in Table 4. Significant differences were found in the frequency of death (*P* = 0.047) and fatal PE (*P* = 0.010) among these phenotypes. The rates of all-cause death (7.4%), fatal PE (5.9%) and major bleeding (7.0%) were all highest in phenotype cluster D, although the difference in major bleeding was not significant. No significant difference was found in the length of stay among these phenotypes.

## Discussion

This study revealed the heterogeneity in clinical presentation between PE patients with and without syncope, and found that various phenotypes existed in PE patients with syncope. To our knowledge, PCA and cluster analysis were applied for the first time to classify PE subjects with syncope. Four phenotypes were identified. In-hospital outcomes were markedly different among patients with similar SBP (phenotypes A, C, and D). In PE patients with syncope, those with high pulse and RR were at higher risk for adverse outcomes.

### Differences in Clinical Presentation Between Patients With and Without Syncope

In our study, 10.4% patients had syncope as an initial symptom, that is more common than 5.5% in the EMPEROR registry (25). In a meta-analysis (26), the overall prevalence of syncope in PE was 16.9%, ranging between 6.8 and 29.9%. We found the rate of syncope in high-risk PE was 37.4%, comparable with 35% in the German registry (8). The difference of symptoms between patients with and without syncope might reflect different clinical phenotypes in PE. Symptoms, including fever, cough, sputum, hemoptysis and pleurisy pain, could be explained by distal thromboembolism and consequent pulmonary infarction. Other symptoms, like syncope, dyspnea, precordial pain and palpitation, might be caused by central and relative massive clot. In a study derived from RIETE registry, 3,391 PE patients without chronic lung disease or heart failure were divided into three groups: patients with pulmonary infarction, isolated dyspnea and circulatory collapse. Patients with pulmonary infarction had a significantly lower mortality rate (5).

In our study, PE patients with syncope exhibited female-predominance, similar with prior reports (16, 19, 27, 28). Sex difference in the function of the autonomic nervous system can be related to syncope (29). Moreover, smaller and stiffer left ventricular in females may lead to a relatively larger reduction in stroke volume and make females more vulnerable to syncope (30). Besides female gender, history of varicose veins was also found to be associated with syncope in patients with PE. In further analysis, we found patients with varicose veins had higher D-Dimer level, that suggests these patients had higher clot burden. Thrombocytopenia was associated with syncope in patients with PE for the same reason since platelet number might be consumptively reduced due to heavy thrombus burden.

We found that the patients with syncope had higher D-Dimer level, higher frequency of S_I_Q_III_T_III_ in ECG and RV/LV ratio > 0.9 in UCG, higher level of estimated pulmonary arterial systolic pressure in UCG, as well as higher frequency of central thrombus in CT pulmonary angiography (CTPA). These variables reflect clot burden directly or indirectly. We also found that more patients had cardiac injury in the syncope group. Similarly, previous studies have reported that PE patients with syncope showed significantly higher cardiac biomarker levels, as well as higher rates of central PE and RVD (21, 26). The presence of syncope might indicate more severe clot burden which causes cardiac injury and RVD, leading to reduced cardiac output and transient hypoperfusion of brain. This explains the higher prevalence of hemodynamical instability in patients with syncope. The present study shows more patients with SBP < 100 mmHg at admission in the syncope group, also supporting this mechanism for syncope.

### Impact of Syncope on Disease Severity and Outcomes of Patients With PE

Consistent with previous studies (16, 26), we found more patients had hemodynamically unstable PE in the syncope group. Though syncope was not associated with in-hospital outcome, patients with syncope were more often treated with thrombolysis, even in normotensive PE. The all-cause mortality was significantly higher in hemodynamically unstable patients without syncope. We speculate less use of systemic thrombolysis is the main reason leading to higher rate of death in these patients. Similarly, a better 30-day survival was only identified in hemodynamically unstable PE patients with syncope (31). The presence of syncope might be regarded as a sign of severe PE leads to more intensive monitoring and more aggressive treatment. Nonetheless, the prognostic role of syncope in PE varies in different studies (9, 32). These inconsistent results might be attributable to the heterogeneity of mechanism for syncope in PE patients.

### Clinical Phenotypes of PE Patients With Syncope

The underlying mechanisms for syncope during PE are not completely understood (7, 12). In acute PE, syncope may occur when the pulmonary vasculature is occluded more than 50%, which causes a sudden drop in cardiac output and temporary cerebral hypoperfusion (6, 33), or due to arrhythmia caused by right ventricular overload (11). The vasovagal reflex leading to neurogenic syncope is another possible reason (6). These different pathophysiological changes may lead to syncope with different outcomes. We notice that SBP < 100 mmHg is particularly rare in phenotype B with more female patients. Therefore, we speculate that the presence of syncope in phenotype B is probably caused by vasovagal reflex and the dysfunction of the autonomic nervous system, instead of sudden drop in cardiac output. Compared with neurogenic syncope, patients with syncope caused by RVD and drop in cardiac output might have more severe disease and poorer prognosis, that was indicated by the difference between phenotypes B and D. The D-Dimer level was markedly higher in phenotype D, that implied higher clot burden in patients with this phenotype. Higher pulse and RR in this phenotype suggested significant cardiopulmonary compensation caused by heavier clot burden.

Few studies have used cluster analysis to assess phenotypes in patients with PE. In a retrospective study, 551 PE patients were classified into five clusters based on 10 symptoms, using PCA and system clustering method (34). However, they did not compare the outcome among different phenotypes. We focused on the patients with syncope and tried to identify clinically based phenotypes that can be used in daily practice. In the four phenotypes identified, phenotype D consisted of patients with high pulse and RR had the poorest outcome. Although no significant difference was found in the prevalence of SBP < 100 mmHg among phenotypes A, C, and D, outcomes during admission were markedly different. Pulse and RR might be more sensitive than SBP in the assessment of disease severity in acute PE.

## Limitations

There are some limitations in our study. Firstly, syncope remains a symptom difficult to define, frequently reported by relatives or bystanders. It may lead to imprecise estimates of its prevalence as well as the associated risks among patients with PE. Secondly, in this longstanding, multicenter registry study, ultrasonic or radiological parameters related to RVD are missing for some patients, which made further analysis difficult. In addition, our phenotyping was exclusively based on clinical variables, which could be improved with the inclusion of variables relevant to the pathogenesis of syncope, such as imaging-derived parameters associated with RVD and cardiac biomarkers. Further studies are needed to validate the prognostic value of these phenotypes and illustrate the underlying pathophysiological mechanisms leading to a particular phenotype.

## Conclusion

In summary, we found differences in the clinical characteristics of PE patients with and without syncope. Syncope was associated with hemodynamic instability and more application of thrombolysis, whereas it did not impact the in-hospital outcomes. We identified four phenotypes with prognostic implications in PE patients with syncope. PE patients with syncope need to be managed more cautiously if they had higher pulse and RR, since they are at high risk for adverse prognosis.

## Data Availability Statement

The original contributions presented in the study are included in the article/Supplementary Material, further inquiries can be directed to the corresponding authors.

## Ethics Statement

The studies involving human participants were reviewed and approved by the Ethic Committee of all participating centers (Approval No. 2012BJYYEC-050-02, 2017-24). The participants provided their written informed consent to participate in this study.

## Author Contributions

ZheZ and CW had full access to all of the data in the study, take responsibility for the content of manuscript, and conceived and designed the study. SZ, XX, YJ, YY, QY, HC, XH, ZL, and YM analyzed the data and drafted the manuscript. JL, DW, ZhuZ, and SW integrated the data and take responsibility for the accuracy of the data analysis. YZ, MZ, and XS participated in data acquisition. JZ, JS, QG, XT, WX, JW, ZhoZ, BF, and PY contributed to the clinical inputs and interpretation of the data. All authors provided final approval of the version to be published.

## Funding

This work was supported by CAMS Innovation Fund for Medical Sciences (CIFMS) (No. 2021-I2M-1-049), the National Key R&D Program of China, Ministry of Science and Technology of China (No. 2018YFC1315100), the Fund of the National Key Research and Development Program of China (No. 2016YFC0905600), and Elite Medical Professionals Project of China-Japan Friendship Hospital (No. ZRJY2021-QM11).

## Conflict of Interest

The authors declare that the research was conducted in the absence of any commercial or financial relationships that could be construed as a potential conflict of interest.

## Publisher's Note

All claims expressed in this article are solely those of the authors and do not necessarily represent those of their affiliated organizations, or those of the publisher, the editors and the reviewers. Any product that may be evaluated in this article, or claim that may be made by its manufacturer, is not guaranteed or endorsed by the publisher.
