# Supplementary material for: Clinical Phenotypes With Prognostic Implications in Pulmonary Embolism Patients With Syncope
Source: Front Cardiovasc Med. 2022 Feb 15;9:836850. doi: 10.3389/fcvm.2022.836850 (PMC8886035; doi:10.3389/fcvm.2022.836850)
Supplement: Supplementary file 1 [file Table_1.docx]

**SUPPLEMENTARY MATERIAL**

**Table S1. Electrocardiogram, cardiac ultrasonography and CTPA findings of patients with acute PE.**

| **Characteristics** | **Patients with Syncope**  **(n=777)** | **Patients without Syncope**  **(n=6661)** | ***P* value** |
| --- | --- | --- | --- |
| **Electrocardiogram findings, n (%)** |  |  |  |
| S_I_ | 178 (25.6) | 871 (16.1) | <0.001* |
| Q_III_ | 215 (30.9) | 943 (17.4) | <0.001* |
| T_III_ | 222 (31.9) | 1000 (18.4) | <0.001* |
| RV Hypertrophy | 26 (3.7) | 151 (2.8) | 0.156 |
| Pulmonary wave | 23 (3.3) | 143 (2.6) | 0.304 |
| **Cardiac ultrasonography findings** |  |  |  |
| RV/LV ratio > 0.9, n (%) | 67 (50.0) | 298 (29.6) | <0.001* |
| RV free wall mobility ≤ 5mm, n (%) | 54 (58.7) | 281 (37.7) | <0.001* |
| Estimated pulmonary arterial systolic pressure, mmHg | 51 (40, 66) | 47 (35, 63) | 0.001* |
| **Thrombus location in CTPA, n (%)** |  |  |  |
| Main pulmonary artery | 138 (20.8) | 544 (10.0) | <0.001* |
| Right pulmonary artery | 387 (57.7) | 2033 (37.0) | <0.001* |
| Left pulmonary artery | 319 (47.9) | 1501 (27.3) | <0.001* |

Abbreviations: CTPA, CT pulmonary angiography; PE, pulmonary embolism; RV, right ventricular; LV, left ventricular.

* The difference is statistically significant**.**
