# Supplementary material for: Clinical Phenotypes With Prognostic Implications in Pulmonary Embolism Patients With Syncope
Source: Front Cardiovasc Med. 2022 Feb 15;9:836850. doi: 10.3389/fcvm.2022.836850 (PMC8886035; doi:10.3389/fcvm.2022.836850)
Supplement: Supplementary file 2 [file Table_2.docx]

**SUPPLEMENTARY MATERIAL**

**Table S2. Comparison of demographic characteristics, comorbid diseases and clinical presentation between patients with and without syncope in hemodynamically stable group and hemodynamically unstable group.**

| **Characteristics** | **Hemodynamically stable PE patients**  **(n=7128)** | | | | | **Hemodynamically unstable PE patients**  **(n=310)** | | |
| --- | --- | --- | --- | --- | --- | --- | --- | --- |
|  | **Patients with Syncope**  **(n=661, 9.3%)** | | **Patients without Syncope**  **(n=6467, 90.7%)** | ***P* value** | **Patients with Syncope**  **(n=116, 37.4%)** | | **Patients without Syncope**  **(n=194, 62.6%)** | ***P* value** |
| **Demographic characteristics** | |  |  |  |  | |  |  |
| Age, year, mean(SD) | | 60.10 (14.94) | 61.43 (15.09) | 0.031* | 60.02 (14.63) | | 60.10 (14.85) | 0.969 |
| Male, n(%) | | 286 (43.3) | 3497 (54.1) | <0.001* | 50 (43.1) | | 106 (54.6) | 0.049* |
| BMI, kg/m^2^, mean(SD) | | 24.41 (3.51) | 24.05 (3.60) | 0.018* | 24.30 (3.73) | | 23.68 (3.93) | 0.178 |
| **Comorbid diseases, n(%)** | |  |  |  |  | |  |  |
| Cardiovascular diseases | |  |  |  |  | |  |  |
| Hypertension | | 255 (38.6) | 2307 (35.7) | 0.140 | 41 (35.3) | | 67 (34.5) | 0.885 |
| Coronary heart disease | | 82 (12.4) | 881 (13.6) | 0.381 | 14 (12.1) | | 27 (13.9) | 0.642 |
| Rheumatic heart disease | | 4 (0.6) | 44 (0.7) | 1.000 | 0 | | 0 | - |
| Cardiomyopathy | | 3 (0.5) | 40 (0.6) | 0.797 | 0 | | 2 (1.0) | 0.530 |
| Heart failure | | 14 (2.1) | 333 (5.2) | 0.001* | 4 (3.4) | | 11 (5.7) | 0.543 |
| Respiratory diseases | |  |  |  |  | |  |  |
| Chronic obstructive pulmonary disease | | 34 (5.2) | 544 (8.4) | 0.003* | 4 (3.4) | | 16 (8.2) | 0.154 |
| Pulmonary infection | | 36 (5.5) | 797 (12.3) | <0.001* | 11 (9.5) | | 16 (8.2) | 0.709 |
| Tuberculosis | | 18 (2.7) | 202 (3.1) | 0.570 | 2 (1.7) | | 4 (2.1) | 1.000 |
| Asthma | | 3 (0.5) | 89 (1.4) | 0.045* | 3 (2.6) | | 1 (0.5) | 0.297 |
| Interstitial lung disease | | 8 (1.2) | 116 (1.8) | 0.274 | 1 (0.9) | | 5 (2.6) | 0.526 |
| Bronchiectasis | | 4 (0.6) | 83 (1.3) | 0.184 | 0 | | 4 (2.1) | 0.301 |
| Cor pulmonale | | 21 (3.2) | 163 (2.5) | 0.311 | 0 | | 5 (2.6) | 0.161 |
| Diabetes mellitus | | 63 (9.6) | 694 (10.8) | 0.350 | 13 (11.3) | | 25 (13.0) | 0.670 |
| Neurological diseases | |  |  |  |  | |  |  |
| Ischemic stroke | | 58 (8.8) | 564 (8.8) | 0.966 | 8 (6.9) | | 14 (7.2) | 0.915 |
| Hemorrhagic stroke | | 9 (1.4) | 125 (1.9) | 0.302 | 1 (0.9) | | 4 (2.1) | 0.730 |
| Chronic hepatitis | | 7 (1.1) | 114 (1.8) | 0.183 | 3 (2.6) | | 3 (1.5) | 0.828 |
| Cirrhosis | | 4 (0.6) | 21 (0.3) | 0.413 | 1 (0.9) | | 2 (1.0) | 1.000 |
| Chronic nephritis | | 3 (0.5) | 63 (1.0) | 0.265 | 2 (1.7) | | 2 (1.0) | 0.997 |
| Nephrotic syndrome | | 7 (1.1) | 72 (1.1) | 0.901 | 2 (1.7) | | 4 (2.1) | 1.000 |
| Varicose veins | | 58 (8.8) | 453 (7.0) | 0.090 | 17 (14.7) | | 13 (6.7) | 0.022* |
| **Risk factors for PE, n(%)** | |  |  |  |  | |  |  |
| Malignancy | | 56 (8.5) | 805 (12.5) | 0.003* | 13 (11.2) | | 25 (12.9) | 0.663 |
| Surgery in recent 3 months | | 92 (13.9) | 858 (13.3) | 0.642 | 21 (18.1) | | 37 (19.1) | 0.832 |
| Trauma in recent 3 months | | 46 (7.0) | 513 (8.0) | 0.361 | 15 (12.9) | | 28 (14.4) | 0.711 |
| Central venous catheterization | | 5 (0.8) | 35 (0.6) | 0.483 | 0 | | 2 (1.0) | 0.529 |
| Oral contraceptives | | 5 (1.3) | 19 (0.6) | 0.134 | 2 (3.0) | | 0 | 0.182 |
| Pregnancy | | 3 (0.8) | 76 (2.6) | 0.053 | 0 | | 4 (4.5) | 0.135 |
| Postpartum | | 61 (16.3) | 502 (16.9) | 0.757 | 12 (18.2) | | 21 (23.9) | 0.395 |
| **Ever or current smoker, n(%)** | | 161 (31.4) | 2000 (37.6) | 0.006* | 31 (27.9) | | 67 (37.2) | 0.103 |
| **Symptoms, n(%)** | |  |  |  |  | |  |  |
| Cough | | 199 (30.1) | 2827 (43.8) | <0.001* | 21 (18.1) | | 61 (31.4) | 0.010* |
| Sputum | | 155 (23.4) | 2174 (33.7) | <0.001* | 16 (13.8) | | 48 (24.7) | 0.021* |
| Fever | | 56 (8.5) | 1037 (16.1) | <0.001* | 4 (3.4) | | 28 (14.4) | 0.004* |
| Dyspnea | | 467 (70.7) | 4309 (66.8) | 0.044* | 74 (63.8) | | 143 (73.7) | 0.065 |
| Precordial pain | | 208 (31.5) | 1542 (23.9) | <0.001* | 35 (30.2) | | 52 (26.8) | 0.523 |
| Pleurisy pain | | 85 (12.9) | 1172 (18.2) | 0.001* | 8 (6.9) | | 21 (10.8) | 0.250 |
| Hemoptysis | | 56 (8.5) | 921 (14.3) | <0.001* | 6 (5.2) | | 22 (11.3) | 0.067 |
| Palpitation | | 170 (25.7) | 768 (11.9) | <0.001* | 23 (19.8) | | 39 (20.1) | 0.953 |
| **Signs** | |  |  |  |  | |  |  |
| Temperature, ℃ | | 36.5 (36.2, 36.8) | 36.5 (36.3, 36.9) | <0.001* | 36.5 (36.2, 36.8) | | 36.5 (36.1, 36.8) | 0.256 |
| Pulse, beats/min | | 87 (78, 98.5) | 84 (76, 96) | <0.001* | 91 (80, 110) | | 95 (80, 110) | 0.538 |
| Respiratory rate, times/min | | 20 (19, 22) | 20 (18, 22) | 0.008* | 21 (19, 24) | | 22 (20,25) | 0.097 |
| Systolic blood pressure, mmHg | | 126 (112, 140) | 126 (116, 140) | 0.074 | 109.5 (94.0, 129.3) | | 100.5 (85.0, 126.0) | 0.018* |
| Diastolic blood pressure, mmHg | | 80 (70, 87.75) | 80 (70, 85) | 0.937 | 70.0 (60.0, 81.3) | | 66.0 (55.5, 78.5) | 0.134 |
| **Laboratory findings** | |  |  |  |  | |  |  |
| WBC, ×10^9^/L | | 8.23 (6.13, 10.59) | 7.65 (5.90, 10.10) | 0.002* | 9,63 (7.30, 11.96) | | 9.42 (7.14, 11.83) | 0.742 |
| HGB, g/L, mean(SD) | | 129.13 (20.65) | 128.15 (21.51) | 0.266 | 125.55 (21.10) | | 125.78 (24.49) | 0.935 |
| PLT, ×10^9^/L | | 188 (147.5, 235) | 202 (157, 253) | <0.001* | 205.5 (148.3, 266.0) | | 188.0 (140.3, 258.8) | 0.300 |
| PaO_2_<60mmHg, n(%) | | 124 (20.6) | 1115 (20.2) | 0.809 | 33 (30.6) | | 45 (27.6) | 0.600 |
| eGFR<60ml/min/1.73m^2^, n(%) | | 98 (15.6) | 777 (12.6) | 0.038* | 24 (21.2) | | 54 (29.7) | 0.110 |
| Elevated cardiac biomarkers, n(%) | | 335 (50.7) | 2234 (34.5) | <0.001* | 75 (64.7) | | 94 (48.5) | 0.006* |

Abbreviations: PE, pulmonary embolism; SD, standard deviation; BMI, body mass index; WBC, white blood cell; HGB, hemoglobin; PLT, platelet; eGFR, estimated glomerular filtration rate, assessed by CKD-EPI formula.

* The difference is statistically significant.
