# Supplementary material for: Clinical Phenotypes With Prognostic Implications in Pulmonary Embolism Patients With Syncope
Source: Front Cardiovasc Med. 2022 Feb 15;9:836850. doi: 10.3389/fcvm.2022.836850 (PMC8886035; doi:10.3389/fcvm.2022.836850)
Supplement: Supplementary file 3 [file Table_3.docx]

**SUPPLEMENTARY MATERIAL**

**Table S3. Comparison of initial treatment and in-hospital outcome between patients with and without syncope in hemodynamically stable group and hemodynamically unstable group.**

| **Characteristics** | | **Hemodynamically stable PE patients**  **(n=7128)** | | | **Hemodynamically unstable PE patients**  **(n=310)** | | |
| --- | --- | --- | --- | --- | --- | --- | --- |
|  | **Patients with Syncope**  **(n=661, 9.3%)** | | **Patients without Syncope**  **(n=6467, 90.7%)** | ***P* value** | **Patients with Syncope**  **(n=116, 37.4%)** | **Patients without Syncope**  **(n=194, 62.6%)** | ***P* value** |
| **Initial treatment, n(%)** |  | |  |  |  |  |  |
| Anticoagulation | 518 (78.4) | | 5567 (86.1) | <0.001* | 45 (38.8) | 97 (50.0) | 0.055 |
| Systemic thrombolysis | 106 (16.0) | | 461 (7.1) | <0.001* | 60 (51.7) | 68 (35.1) | 0.004* |
| Inferior vena cava filter | 19 (3.0) | | 341 (5.4) | 0.005* | 14 (12.5) | 12 (6.3) | 0.062 |
| Interventional thrombectomy | 2 (0.3) | | 21 (0.3) | 1.000 | 2 (1.8) | 1 (0.5) | 0.638 |
| Surgical embolectomy | 9 (1.4) | | 42 (0.7) | 0.042* | 2 (1.8) | 3 (1.6) | 1.000 |
| **In-hospital outcomes, n(%)** |  | |  |  |  |  |  |
| All-cause death | 13 (2.0) | | 192 (3.0) | 0.177 | 11 (9.5) | 38 (19.6) | 0.018* |
| Fatal PE | 6 (0.9) | | 77 (1.2) | 0.702 | 10 (8.6) | 25 (12.9) | 0.251 |
| Major bleeding | 9 (2.1) | | 92 (2.1) | 0.941 | 3 (3.0) | 9 (5.7) | 0.489 |
| **Days of hospitalization, day** | 13 (9, 13) | | 14 (9, 19) | 0.783 | 13 (9, 20) | 14 (8.5, 20.5) | 0.871 |

Abbreviations: PE, pulmonary embolism.

*The difference is statistically significant.
